# Improving the Robustness of Variable Selection and Predictive Performance of Regularized Generalized Linear Models and Cox Proportional Hazard Models

**DOI:** 10.3390/math11030557

**Published:** 2023-01-20

**Authors:** Feng Hong, Lu Tian, Viswanath Devanarayan

**Affiliations:** 1 Takeda Pharmaceuticals, Cambridge, MA 02139, USA; 2 Department of Biomedical Data Science, Stanford University, Stanford, CA 94305, USA; 3 Eisai Inc., Nutley, NJ 07110, USA; 4 Department of Mathematics, Statistics, and Computer Science, University of Illinois Chicago, Chicago, IL 60607, USA

**Keywords:** variable selection, lasso, elastic-net, Monte Carlo, resampling, cross-validation, predictive modeling, 62J07

## Abstract

High-dimensional data applications often entail the use of various statistical and machine-learning algorithms to identify an optimal signature based on biomarkers and other patient characteristics that predicts the desired clinical outcome in biomedical research. Both the composition and predictive performance of such biomarker signatures are critical in various biomedical research applications. In the presence of a large number of features, however, a conventional regression analysis approach fails to yield a good prediction model. A widely used remedy is to introduce regularization in fitting the relevant regression model. In particular, a $L_{1}$ penalty on the regression coefficients is extremely useful, and very efficient numerical algorithms have been developed for fitting such models with different types of responses. This $L_{1}$-based regularization tends to generate a parsimonious prediction model with promising prediction performance, i.e., feature selection is achieved along with construction of the prediction model. The variable selection, and hence the composition of the signature, as well as the prediction performance of the model depend on the choice of the penalty parameter used in the $L_{1}$ regularization. The penalty parameter is often chosen by K-fold cross-validation. However, such an algorithm tends to be unstable and may yield very different choices of the penalty parameter across multiple runs on the same dataset. In addition, the predictive performance estimates from the internal cross-validation procedure in this algorithm tend to be inflated. In this paper, we propose a Monte Carlo approach to improve the robustness of regularization parameter selection, along with an additional cross-validation wrapper for objectively evaluating the predictive performance of the final model. We demonstrate the improvements via simulations and illustrate the application via a real dataset.

## 1. Introduction

There is a need to develop a prediction model in many biomedical applications, i.e., finding a function of a set of features, which could be biomarkers and patient characteristics, to predict the outcome of interest such as disease status and survival time.

Consider a typical predictive modeling problem, with Y being the binary outcome, indicating cardiovascular death in the next 5 years, and x being a set of baseline covariates such as systolic blood pressure, body mass index, etc. The objective is to predict the value of the binary outcome Y, i.e., if the patient would die from a cardiovascular cause in the next 5 years, or the 5-year cardiovascular risk P(Y=1∣X=x) for a future observation based on his/her covariate x. To this end, we may consider a probability model characterizing the conditional probability P(Y=1∣X=x). The logistic regression model

(1) $$P(Y=1|X=x)=\frac{\text{exp}\;(\beta_{0}+x^{T}\beta )}{1+\text{exp}(\beta_{0}+x^{T}\beta )}$$

is one of the most convenient choices. When an estimator for the regression coefficients ($\beta_{0}$, β), (${\hat{\beta }}_{0}$, $\hat{\beta }$), is obtained, one may predict a future Y based on the estimated conditional probability:

(2) $$\hat{P}(Y=1|X=x)=\frac{\text{exp}\;({\hat{\beta }}_{0}+x^{T}\hat{\beta })}{1+\text{exp}({\hat{\beta }}_{0}+x^{T}\hat{\beta })}$$

When the dimension of x is big, the regularization method is needed to fit the regression model. Lasso, or the $l_{1}$ penalty [1], gained its popularity due to its ability to automatically obtain a sparse solution for settings with high-dimensional covariate X, whose dimension p >> N. As the coefficients of unimportant predictors are shrunk to zero, variable selection is achieved automatically within the model-fitting procedure. An additional ridge penalty is introduced in the elastic-net regularization to accommodate correlated predictors, so that when several strongly correlated predictors are associated with the outcome, the elastic net model will obtain a solution where the group of strongly correlated predictors are selected together, whereas lasso tends to select only one predictor from the cluster and ignores the rest [2]. The elastic-net (lasso as a special case) is implemented in the R package ‘glmnet’. By using cyclical coordinate descent, this numerical algorithm is very fast and efficient [3,4]. Coupled with the appropriate regression model, the elastic-net penalty can be used to handle multiple types of outcomes such as continuous, binary, multinomial, and survival time [5]. Hereafter in this manuscript, we refer to this algorithm as ‘glmnet’.

There is a tuning parameter λ in lasso or elastic-net regularization. It controls the degree of regularization and indirectly determines the number of nonzero coefficients, and hence the number of predictors in the final prediction model (i.e., biomarker signature). In a lasso model, the selection of the tuning parameter is approximately equivalent to the selection of optimal numbers of predictors used in the final prediction model. A smaller λ corresponds to a more complex model with many predictors, and thus may overfit the response in the training set. Conversely, a larger λ corresponds to a more parsimonious model that may underfit the response in the training set. The tuning parameter is selected via cross-validation (CV) to optimize the estimated predictive performance [6,7]. The R package ‘glmnet’ provides a function ‘cv.glmnet’ to conduct K-fold cross-validation, which produces estimates of the prediction performance measured by the root-mean-squared error (RMSE) for continuous outcomes, area under the curve (AUC) for binary outcomes, or deviance for all types of outcomes corresponding to different tuning parameters. The final model is fitted by selecting the λ that achieves the best CV performance ($\lambda_{opt}$).

**Figure 1.**
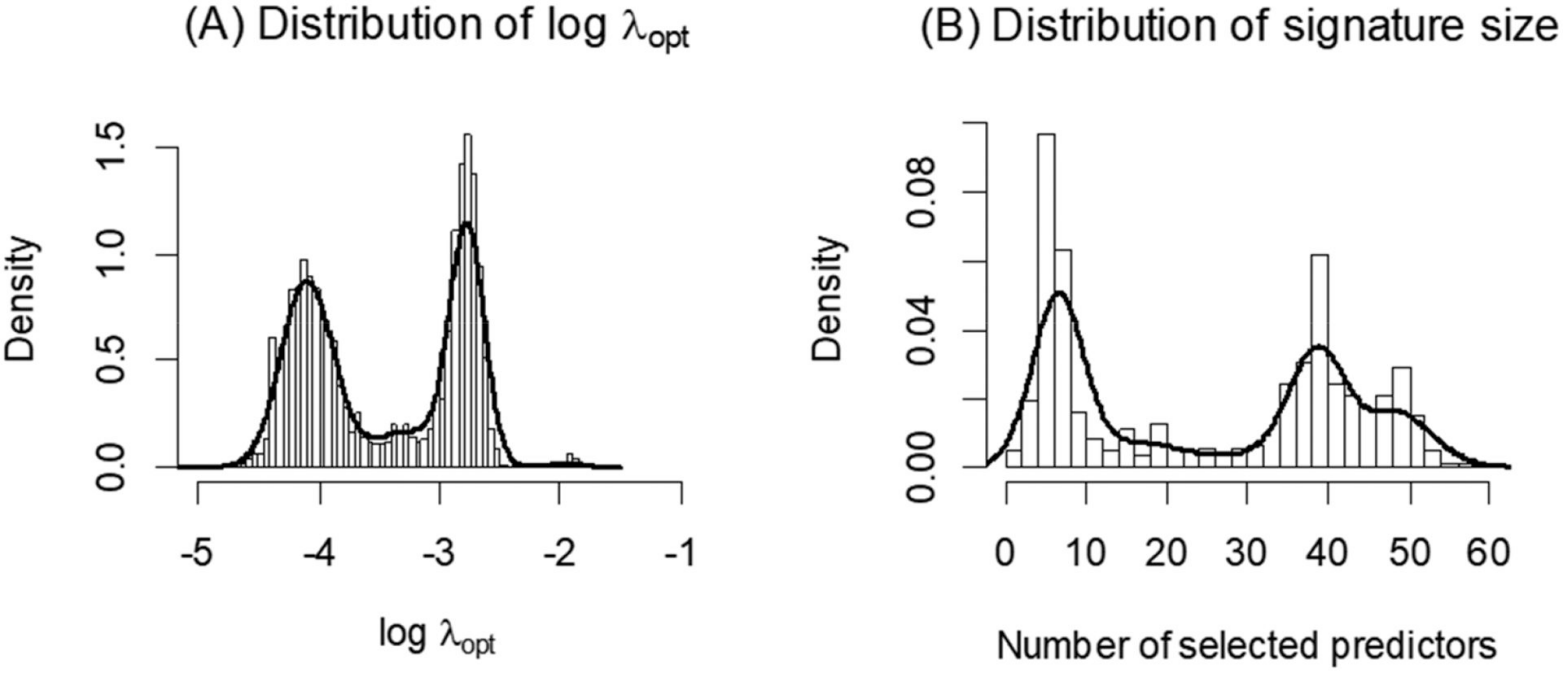
Distribution of $\lambda_{opt}$ (**A**) across 1000 runs of glmnet is shown, along with the distribution of the corresponding number of predictors in the signatures (**B**). Each application of the glmnet algorithm for this dataset results in an optimal signature, with size varying greatly from less than 5 to over 50 predictors.

In our experience, we have observed considerable instability in the selected value of $\lambda_{opt}$ via cross-validation, and hence in the selection of optimal variables/predictors in some practical applications, especially for smaller datasets. This may be due to random variation in the cross-validation procedure. We illustrate this phenomenon using proteomics data from the Alzheimer’s Disease Neuroimaging Initiative (ADNI) dataset [8,9], which includes measurements of 320 biomarkers (peptides) in 135 subjects with mild cognitive impairment (MCI) and 86 age-matched normal subjects (NL). We apply glmnet to identify an optimal combination of biomarkers for classifying NL and MCI subjects. Figure 1A shows the distribution of $\lambda_{opt}$ estimates from 1000 independent iterations of the glmnet algorithm, where a different random seed value was used in each cross-validation. The distribution of the number of selected biomarkers corresponding to the estimated $\lambda_{opt}$ is shown in Figure 1B. As evident from these figures, there is considerable variation in the $\lambda_{opt}$ estimates each time the cross-validation procedure is employed, and thus the signature composition (selected informative biomarkers) and its size (number of selected biomarkers) vary considerably (from fewer than 5 biomarkers to greater than 50 biomarkers) across each application of the ‘cv.glmnet’. This instability makes the applicability of the glmnet algorithm and the scientific interpretation of the peptides included in the resulting predictive signature challenging. It is known that the result from cross-validation procedure depends on the specific data training and testing splitting in the cross-validation, and thus may vary from cross-validation to cross-validation. However, there is oftentimes a misconception that the result from k-fold cross-validation is much more stable due to the fact that every observation has served as a testing data point once and has contributed to the model training k−1 times. The aforementioned simple example shows that stability still can be a serious concern for k-fold cross-validation, whose selected optimal penalty parameter can have a multimodal distribution resulting in quite different choices of $\lambda_{opt}$ depending on the particular implementation of cross-validation. To address this concern, in this paper, we propose a simple Monte Carlo approach for glmnet, which we call MCglmnet. We demonstrate a significant improvement in stability via simulation studies and illustrate it using the ADNI dataset described above.

In addition, we propose the use of the outside cross-validation wrapper on the glmnet and MCglmnet algorithms to evaluate and report the predictive performance of the resulting signatures. We emphasize this because there may be a temptation to use only the performance estimates from the built-in cross-validation procedure within glmnet for selecting $\lambda_{opt}$ (cv.glmnet) as the final performance measure of the signature. However, this estimate tends to be overly optimistic, and the size of the optimistic bias may depend on the aforementioned variability in the selected $\lambda_{opt}$ via cross-validation. In the simulations, we demonstrate a reduction in bias in predictive performance estimates when using the additional outside cross-validation wrapper.

## 2. Methods

### 2.1. MCglmnet Algorithm

Consider a typical predictive modeling problem with N observations and p predictiors. Specifically, let Y denote the the binary outcome and X be the baseline covariates. A logistic regression model assumes Equation (1) and the elastic-net regularization estimates the model parameter by solving the following regularized optimization problem:

(3) $$\underset{\beta_{0},\beta }{\text{min}}R_{\lambda }(\beta_{0},\beta )=\underset{\beta_{0},\beta }{\text{min}}\left[\frac{1}{N}\sum_{i=1}^{N}\left\{y_{i}\left(\beta_{0}+x_{i}^{T}\beta \right)-\log\left(1+\text{exp}\left(\beta_{0}+x_{i}^{T}\beta \right)\right)\right\}+\lambda P_{\alpha }(\beta )\right]$$

where {$(x_{i},y_{i}),\;i=1,\cdots ,n$} is the observed data consisting of n independent identically distributed copies of (X, Y),

(4) $$P_{\alpha }(\beta )=(1-\alpha )\frac{1}{2}{\left\|\beta \right\|}_{l2}^{2}+\alpha {\left\|\beta \right\|}_{l1}=\sum_{j=1}^{p}\left[\frac{1}{2}(1-\alpha )\beta_{j}^{2}+\alpha |\beta_{j}|\right].$$

$P_{\alpha }$ is the elastic-net penalty, which is a weighted average of the ridge regression penalty (α=0) and the lasso penalty (α=1). Here, λ is the penalty parameter controlling the complexity of the final model and is selected via k fold cross-validation in ‘glmnet’. The resulting penalty parameter depends on randomly splitting the dataset into K nonoverlapping parts and may be instable, as discussed in the Introduction section.

We propose to address the stability problem via MCglmnet, where the tuning parameter estimation procedure is repeated multiple (s) times (e.g., s=100), wherein the tuning parameter, $\lambda_{opt}^{\left(t\right)}$ is estimated in each of the s iterations (t=1,…,s) via internal k-fold cross-validation (cv.glmnet), and the final estimate of the tuning parameter, $\lambda_{opt}$, is defined as the median of the $s\lambda_{opt}^{\left(t\right)}$ values. That is, the tuning parameter in MCglmnet algorithm is estimated by

(5) $$median\left\{\lambda_{opt}^{\left(1\right)},\;\ldots \;,\lambda_{opt}^{\left(s\right)}\right\}$$

where $\lambda_{opt}^{\left(t\right)}$ denotes the tuning parameter estimated via k-fold cross-validation in the tth iteration (t=1,…,s). Note here that MCglmnet with s=1 is equivalent to glmnet.

**Remark 1.** *When* s *is large, the empirical distribution of* $\lambda_{opt}^{\left(1\right)},\ldots ,\lambda_{opt}^{\left(s\right)}$ *is a good approximation to that of* ${\hat{\lambda }}_{opt}|$ *data, where the randomness arises from the random splitting of the observed data into* k *folds in the cross-validation procedure. Therefore,* $\lambda_{opt}^{\left(t\right)}$ *is a single random realization and a very crude approximation to the “center” of the conditional distribution* (${\hat{\lambda }}_{opt}|$*data), which is better estimated by the median of* $\lambda_{opt}^{\left(1\right)},\ldots ,\lambda_{opt}^{\left(s\right)}$. *Alternatively, we may choose the penalty parameter to be an estimate of*

(6) $$E({\hat{\lambda }}_{opt}data).$$

The “center” for the distribution of $\lambda_{opt}^{\left(1\right)},\ldots ,\lambda_{opt}^{\left(s\right)}$ (median or mean) is a more stable choice for the penalty parameter than a simple random realization, when the variability of $\lambda_{opt}^{\left(t\right)}$ is not ignorable, as in Figure 1.

**Remark 2.** *Applying a Monte Carlo simulation to stabilize a random quantity is a common approach. In the aforementioned procedure, we can also directly define the prediction error as a function of* λ in each of the Monte Carlo iterations, denoted by

(7) $$l_{{cv}_{s}}(\lambda )=\sum_{i=1}^{k}\sum_{i\in D_{i}^{s}}l(y_{i},{\hat{\beta }}_{\lambda }{\left(D_{-i}^{s}\right)}^{T}x_{i})$$

*where* {$D_{i}^{s},\;i=1,\cdots ,k$} *splits the data into* k *nonoverlapping parts of approximately equal sizes,* $D_{-i}^{s}$ *represents the data not in* $D_{i}^{s}$, ${\hat{\beta }}_{\lambda }(D_{-i}^{s})$ *is the regularized estimator of* β *with penalty parameter* λ *based on data in* $D_{-i}^{s}$, *and* l(⋅,⋅) *is an appropriate loss function. Clearly,* $l_{{cv}_{s}}(\lambda )$ *is also random due to random splits;* $data=D_{1}^{s}\cup D_{2}^{s}\ldots \cup D_{k}^{s}$. *We may choose to stabilize* $l_{{cv}_{s}}(\lambda )$ *first and then determine the penalty parameter. Specifically, the final tuning parameter can be chosen as the minimizer of the loss function*

(8) $$median\{l_{{cv}_{1}}(\lambda ),\cdots ,l_{cvs}(\lambda )\}$$

or

(9) $$S^{-1}\sum_{j=1}^{S}l_{{cv}_{j}}(\lambda ).$$

**Figure 2.**
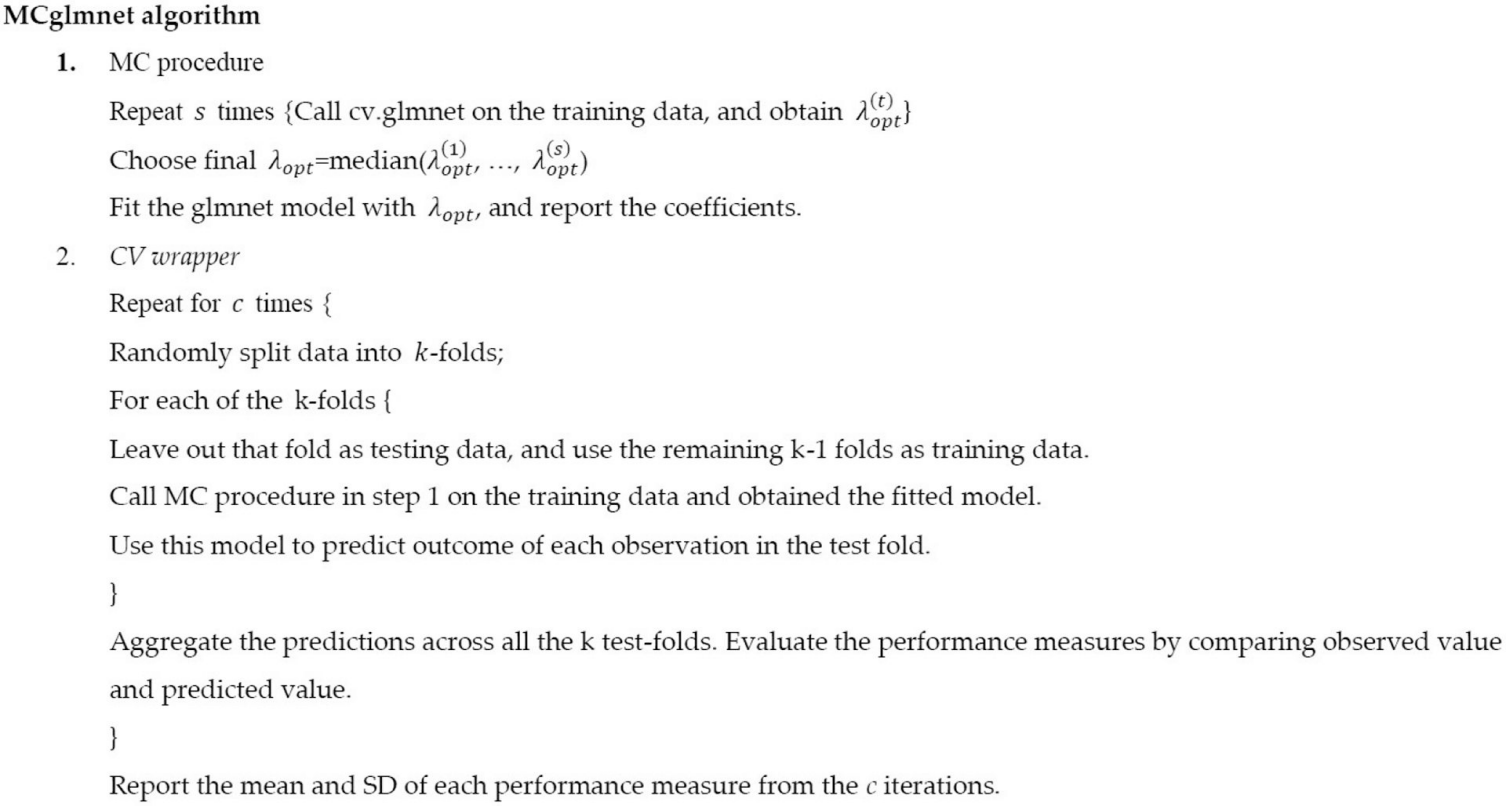
Outline of the MCglmnet algorithm.

To obtain a less biased estimate of the performance of this procedure, another cross-validation (CV wrapper) is conducted around the entire tuning parameter estimation procedure. Furthermore, this cross-validation wrapper is repeated c times (e.g., c=50), and the mean and standard deviation (SD) of each performance measure (e.g., sensitivity, specificity) are reported to summarize the performance of the entire procedure. Figure 2 displays the key steps in the implementation of the MCglmnet algorithm.

**Figure 3.**
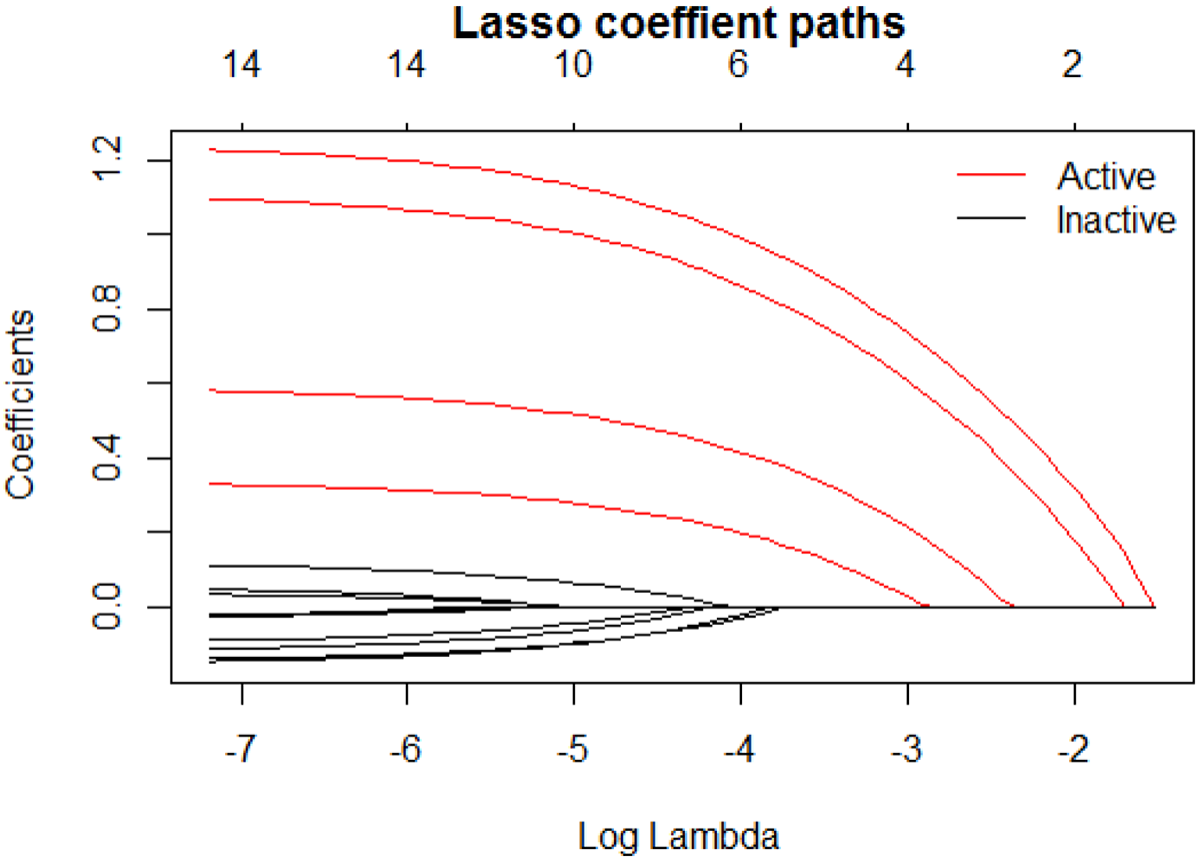
A lasso coefficient solution path.

In a lasso model via glmnet, the selected variables are solely determined by the tuning parameter λ. Figure 3 shows an example of a coefficient solution path for different values of λ. The paths for active variables are highlighted in red. As λ decreases, the order of the variables being added to the model is defined. Once a final λ is estimated via cross-validation, the composition of the signature is determined by the variables with nonzero coefficients. Note that the same solution path is used by MCglmnet and glmnet. The difference between the two approaches is how the final λ is estimated. This means that the stability of variable selection, and hence the signature, depends entirely on the stability of λ estimate. By introducing the Monte Carlo step in the MCglmnet procedure and choosing the final λ estimate to be the median of many replicated runs of the glmnet procedure, the variability of this λ estimate is always smaller than the variability of the λ estimate from a single run of glmnet. Since the solution path used by MCglmnet and glmnet is fixed for a given training dataset, the stability of the number of variables selected (signature size) is equivalent to the stability of the composition of the signature, and thus we can use the variability of the signature size to represent the overall stability of the signature.

In this paper, we describe and present the characteristics of the glmnet and MCglmnet algorithms for a binary outcome, without loss of generality. While there are many different performance measures for a predictive model of a binary outcome such as sensitivity, specificity, accuracy, positive predictive values (PPV), negative predictive value (NPV), area under the receiver operating characteristic curve (AUC), deviance, etc., for the sake of simplicity, we will use only AUC for the tuning parameter selection in the internal cross-validation procedure, and to summarize the predictive performance results from the cross-validation wrapper.

### 2.2. Research Questions

In this section, we address the following three questions via simulation:

1. Does MCglmnet produce more stable signatures than glmnet?
2. Is the predictive performance of MCglmnet better than glmnet?
3. Is an outer cross-validation a better procedure than the built-in cross-validation in evaluating the prediction performance?

### 2.3. Simulation Design

We designed our simulation studies to address the research questions, and to mimic the situations that are typically seen in real data.

The response variable Y was generated from a logistic regression model,

(10) $$\log\left\{\frac{\text{Pr}(Y=1|X=x)}{\text{Pr}(Y=0|X=x)}\right\}=a+b_{1}X_{1}+b_{2}X_{2}+\ldots +b_{5}X_{5}$$

where the five active variables $X_{1}$, $X_{2},\ldots ,X_{5}$ are all randomly generated from N(0,1), and a=0, $\left(b_{1},b_{2},b_{3},b_{4},b_{5})=(8m,\;7m,\;6m,\;5m,\;4m\right)$ and the value of m is 0.04, 0.07, 0.15, which corresponds to the true AUC of 0.7, 0.8, and 0.9, respectively. Here, the true AUC refers to the ROC AUC of the true probability from the generating model versus the ‘observed y’, where the ‘observed $Y^{\prime }$ is from the binomial distribution with the corresponding true probability. The assumed True AUC values, 0.7, 0.8, and 0.9, denote different predictive strengths of the simulation model. We then generate 50, 500, or 5000 inactive variables W (not associated with Y) from N(0,1). In addition to each of these sets of inactive variables, we generate 4, 40, or 400 correlated variables $\tilde{X}$, respectively. Among these 4, 40, and 400 variables, half are generated to have correlations of 0.5 and the other half with correlations of 0.8 with the five informative predictors. For example, the 400 correlated variables are generated such that 80 are correlated with each of the five true predictors, where 40 have correlations of 0.5 and the other 40 have correlations of 0.8. In the case where four correlated variables are generated, we use only the first two true predictors, and generate two correlated variables for each predictor, one with a correlation of 0.5 and the other with 0.8. The inactive variables are introduced to mimic the real data from high-dimensional genomics and proteomics applications, where the true active variables are hidden among numerous noise variables and are often correlated with a group of other variables.

For the training dataset, we consider three different sample size scenarios: n=100, 500, and 1000. Each training set was paired with a test set of sample size N=10,000 that was independently generated in the same way as the corresponding training set. The reason we chose a large test set was to obtain an accurate estimate of the true external predictive performance of the prediction model based on the training set (‘External AUC’), which will be used as an unbiased measure for comparing the performance of MCglmnet and glmnet for different scenarios. In total, 27 scenarios of training and testing datasets were generated (3 levels of signal strength × 3 levels of number of noise variables × 3 training set sample sizes). Each of these simulated datasets were then analyzed using glmnet and MCglmnet, and the results were then compared with respect to the stability of variable selection (size and composition of the signatures) and the predictive performance. Different numbers of MC iterations (s=10, 20, 50, 100, 200, 300, 400, and 500) were considered in this evaluation to obtain insights on the optimal number of iterations that ensures stability of variable selection. To evaluate the stability of the signature, we used the inter-percentile range between the 10th percentile and the 90th percentile (IPR-90) of the signature size obtained from 100 replicated runs of the method on the training datasets for each scenario. The rationale for reporting IPR-90 instead of the inter-quartile range (IQR) is that we wanted to capture the full range of instability of the signature size.

The entire simulation design can be summarized by the following steps:

1. Fix m∈{0.04,0.07,0.15}, $\left(m_{1},m_{2})\;\in \;\{(50,\;4),\;(500,\;40),\;(5000,\;400)\right\}$, and n∈{100,500,1000} (27 combinations).
2. Generate a training set consisting of n copies of a p dimensional covariate vector X and a binary response Y. The following steps are used to generate each individual copy.
  - Generate informative predictor $X_{1},\cdots ,X_{5}$ from standard normal.
  - Generate binary response Y via a logistic regression model with the regression coefficients for ($X_{1},\cdots ,X_{5}$) being (8,7,6,5,4)′×m.
  - Generate $m=m_{1}+m_{2}$ additional predictors:
    - Generate $m_{1}$ noise variables $X_{N}$ from standard normal independent of ($X_{1},\cdots ,X_{5}$).
    - Generate $m_{2}$ correlated variables $X_{C}$ from standard normal but correlated with ($X_{1},\cdots ,X_{5}$) with specified correlation coefficient.
  - Let $X={\left(X_{1},\cdots ,X_{5},X_{N}^{T},\;X_{C}^{T}\right)}^{T}$ and Y=Y.
3. Generate a test set consisting of N=10,000 copies of (X, Y) as in step (2).
4. Data analysis:
  - Apply MCglmnet with s=10, 20, 50, 100, 200, 300, 400, and 500 MC iterations to select the optimal tuning parameter and train a lasso-regularized logistic regression prediction model using the training set.
  - Apply conventional glmnet to select the optimal tuning parameter and train a lasso-regularized logistic regression prediction model using the training set.
  - Apply the outer cross-validation to estimate AUCs using the training set.
  - Apply the prediction model from step (4a) and (4b) to the testing set and calculate the external AUC using the test set.
5. Repeat steps 2–4 100 times and summarize the simulation result by
  - The IPR-90 of the signature sizes from the training sets;
  - The mean of AUC estimates from internal and outer cross-validations from training sets;
  - The mean and SD of the external AUC from test sets.

## 3. Results

### 3.1. Stability of Variable Selection

**Figure 4.**
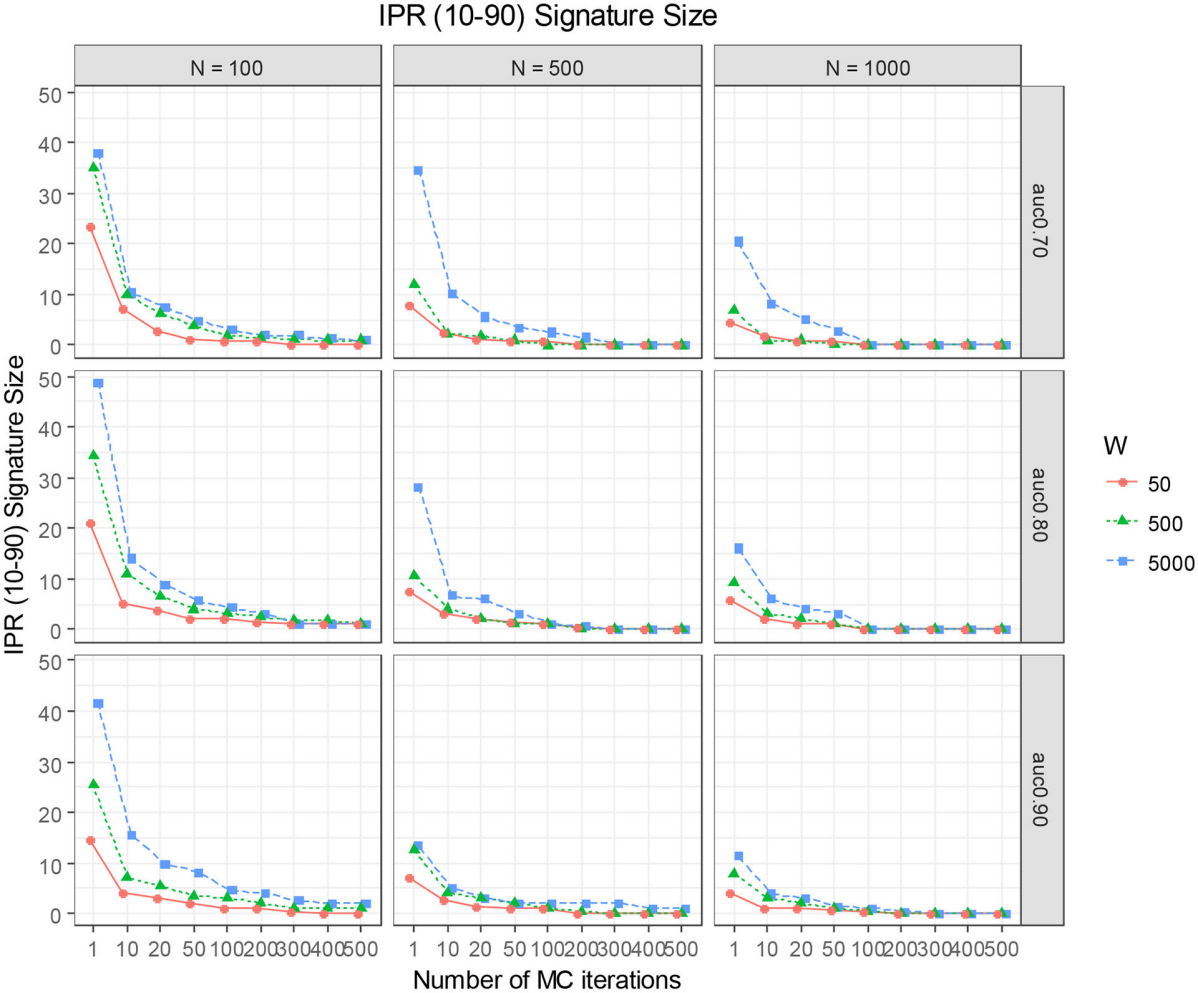
Inter-percentile (10~90%) range (IPR) values of the signature size corresponding to the $\lambda_{opt}$ estimates from 100 replicate runs of glmnet (s = 1) and MCglmnet with different numbers of MC iterations (s = 10, 20, 50, 100, 200, 300, 400, 500) and different levels of signal strength (AUC = 0.7, 0.8, 0.0) are summarized in this graph.

Figure 4 shows the IPR values obtained for all 27 simulation scenarios. For each scenario, variability of the signature size dropped consistently with the increasing number of MC iterations. For most scenarios, 50 MC iterations (i.e., s=50) were adequate to ensure stability of the signature size. Without the MC step iterations (i.e., glmnet; s=1) or with only a few MC iterations, the size of the signature varied considerably for data with moderate sample size (N=100), and this was exacerbated with the increasing number of noise variables. For larger sample sizes (N=500 or 1000), the instability is less severe, but the benefit of the MC iterations is evident in all the scenarios.

### 3.2. Does Mcglmnet Perform Better Than Glmnet?

Having demonstrated considerable improvement in the stability of the signatures with MC iterations of glmnet, the next question of interest is whether MCglmnet performs better than glmnet with respect to the predictive performance. For each of the 27 scenarios in the simulation design, we conducted 100 replicate runs of MCglmnet with s=1 (glmnet) and with s=50 iterations. The mean and SD of the external test set AUC of 100 replicate runs for all these scenarios are summarized in Table 1. It is evident from this table that predictive performance of MCglmnet is mostly similar to that of glmnet, but with lower variability (SD).

### 3.3. Is an Outer Cross-Validation Needed?

In this same table, we also report the bias of the AUC from the internal CV procedure and the outside 5-fold CV wrapper. The AUC values from the internal CV tend to overestimate the performance, as reflected by the positive bias relative to the external test set AUC; this is especially evident when the signal is weak or the sample size is small. This bias arises due to using the same internal CV for both estimating the optimal λ to derive the final signature/model and also to report the predictive performance. The AUC estimates from the outside 5-fold CV wrapper reduced this bias and were closer to the external test set AUC.

Lastly, we have conducted a more comprehensive simulation to cover other simulation scenarios, and the conclusion of the simulation study remains the same. For example, when the correlation between active variables (true predictor) and correlation variables (surrogate to the true predictor) becomes weaker, the stability of variable selection becomes better in general, but more MC iterations can still bring substantial improvement.

## 4. Real Data Application: ADNI Proteomics Data

We applied MCglmnet with s = 50 along with glmnet (MCglmnet with s = 1) to the ADNI proteomics data described in Section 1. The MCglmnet and glmnet algorithms were replicated 100 times to assess and compare the stability of variable selection, and the final signatures were recorded.

**Figure 5.**
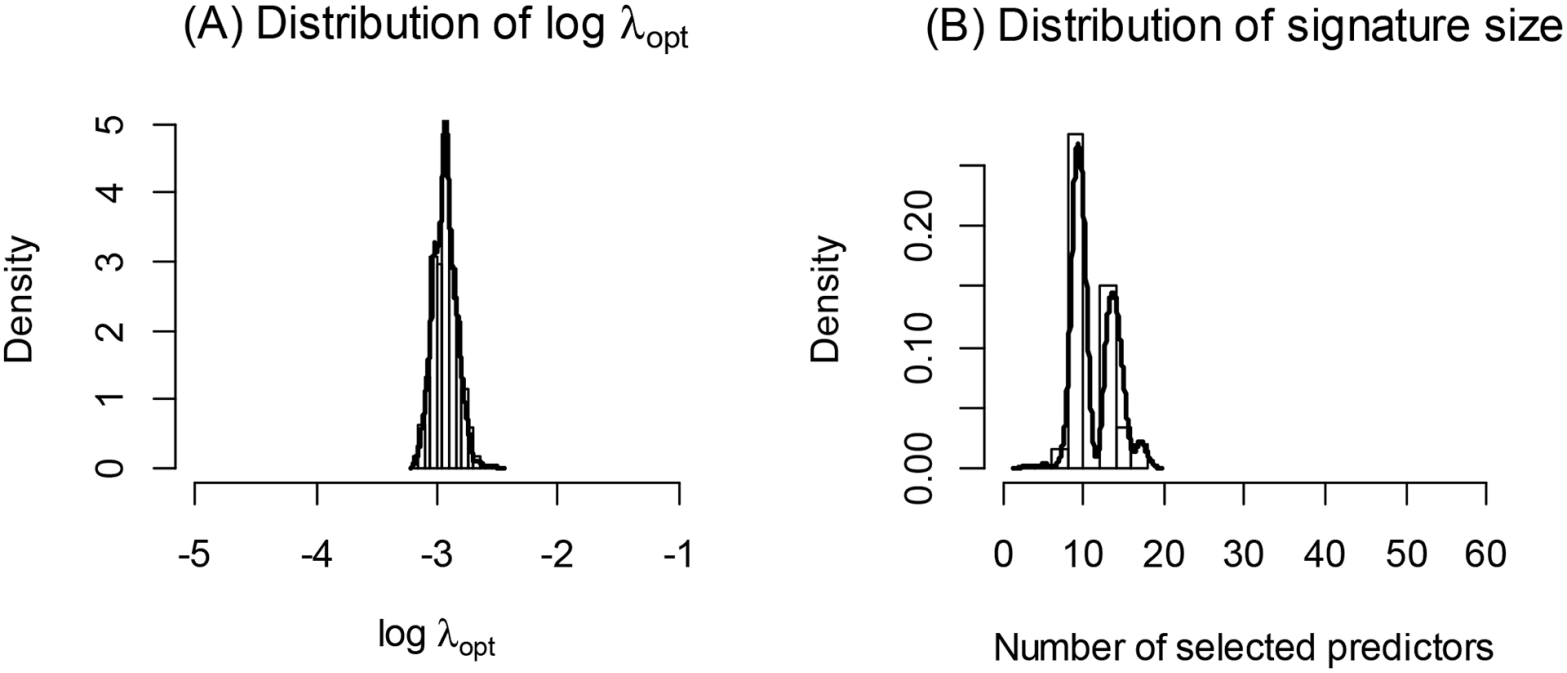
Distribution of $\lambda_{opt}$ (**A**) across 100 replicated runs of MCglmnet (s=50) is shown, along with the distribution of the corresponding number of predictors in the signatures (**B**). Comparison of these distributions to Figure 1 reveals that the signatures derived via MCglmnet are considerably more stable than those derived via glmnet (MCglmnet with s=1).

**Figure 6.**
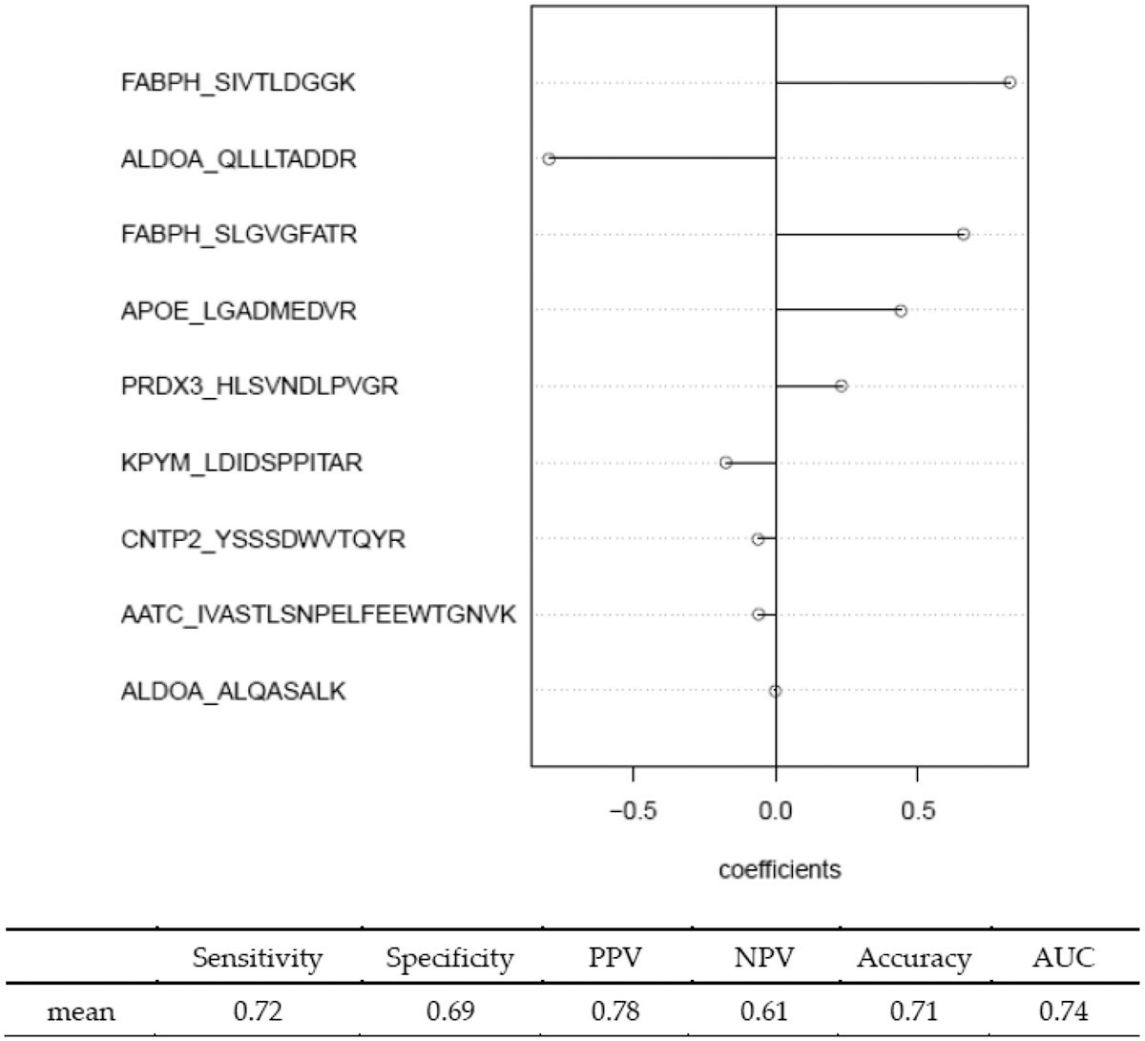
Signature identified by MCglmnet for classifying MCI and NL subjects, along with performance estimates derived via 5-fold outside cross-validation wrapper.

Figure 5A shown the probability density plot of log(λ) estimated from the 100 replicated runs of the MC algorithm. Comparing this to Figure 1, it is evident that the MCglmnet with s = 50 yields a much more stable distribution of log(λ), which translates to more stable variable selection, as shown in Figure 5B and Table 2. The final signature obtained from MCglmnet with s = 50 is based on nine biomarkers, with the standardized coefficients of the biomarkers plotted in Figure 6. The AUC estimated from 10 iterations of 5-fold embedded outer cross-validation is 0.74.

## 5. Discussion

There is an impression that cross-validation is a gold standard in selecting tuning parameters in training a prediction model, and the K-fold cross-validation is quite stable and generates reproducible results. However, the numerical study in this paper shows that there is substantial Monte Carlo variability remaining in the cross-validation method if the size of training and testing splits is small or moderate. This issue can be easily resolved using the proposed method, but may cause serious reproducibility problems if left unaddressed.

Although not reported in this paper, we also obtained the results of MCglmnet and glmnet in which the tuning parameter is chosen to be one standard error ($\lambda_{1se}$) below the $\lambda_{opt}$ generating the optimal CV performance. The use of $\lambda_{1se}$ results in a more parsimonious signature, which tends to have less variability than the signature corresponding to the $\lambda_{opt}$ estimate. However, the predictive peformance of the signature from $\lambda_{1se}$ (as measured by external AUC and outside cross-validation AUC) tends to be lower than the signature from $\lambda_{opt}$. This is not a surprise, considering the tradeoff between the prediction performance and the sparsity of the final model. Therefore, our recommendation is to use MCglmnet with $\lambda_{opt}$ to obtain a signature that has both good stability and optimal predictive peformance.

This idea of introducing Monte Carlo or other resampling methods (such as bootstrap)) to obtain a more stable signature is applicable to any predictive modeling method that utilizes cross-validation or other numerical optimization methods to adaptively select the tuning hyperparameters. Following the same spirit of Bagging [10], we also tried the bootstrap approach in place of Monte Carlo, splitting data into training and testing sets in the MCglmnet algorithm, and found the results to be very similar. To stabilize the tuning parameter selection by increasing the number of MC iterations is indeed a simple yet effective approach. The practical cost is mainly the longer computational time: one needs to train the prediction model a large number of times in multiple runs of cross-validation. Theoretically, as the number of MC iterations goes to infinity, the selected tuning parameter becomes a function of observed data only, and it is interesting to examine its statistical property such as its variance. The answer to this question may help us to choose the appropriate number of MC iterations in practice, which is important, especially when it is time-demanding to train complex prediction models in cross-validation. Ideally, the number of MC iterations should be selected such that the Monte Carlo variance is relatively small in comparison with the intrinsic variance of the optimal tuning parameter as a random statistic, if the number of MC iterations goes to infinity. Those questions warrant future research.

In this paper, while our simulation studies and real data illustrations were provided for a binary response and a logistic regression model with a lasso penalty, the results and conclusions can be generalized for other types of response variables such as multinomial, continuous, and time-to-event, as well as for different penalty functions such as SCAD [11,12].

## 6. Conclusions

In this paper, we proposed a Monte Carlo modification of the glmnet algorithm to improve the stability of variable section. The new approach for the tuning parameter estimation results in a substantially more stable variable selection and hence a more robust prediction signature. While the average predictive performance may be similar between the new method and conventional practice where the tuning parameter is selected based on a single K-fold cross-validation, the predictive performance of the new MCglmnet tends to be more stable (i.e., less variable), especially for small-to-moderate sample size and weak to moderate signal. In addition, the performance estimates from the internal cross-validation procedure used for estimating the tuning parameter in the glmnet algorithm tends to be overly optimistic for smaller datasets with weak-to-moderate signal. This is because the tuning parameter selection is not included as part of the cross-validation. This bias is substantially reduced or eliminated by embedding the tuning parameter estimation within an outside cross-validation wrapper to estimate the performance of the resulting signature.

## Figures and Tables

**Table 1.** Summary of external AUC from 100 replicated runs of each method and the bias of AUC reported by internal CV and the outside 5-fold CV wrapper. For MCglmnet, s = 50.

Simulated Dataset				Glmnet (s = 1) External AUC		MCglmnet (s = 50) External AUC		Bias of AUC Reported by Internal CV †	Bias of AUC Reported by Outside 5-fold CV ‡
Scenario	True AUC	N	W	Median	MAD	Median	MAD		
1	0.7	100	50	58.7%	0.5%	58.8%	0.0%	3.7%	1.0%
2			500	54.1%	0.4%	54.1%	0.0%	2.8%	2.1%
3			5000	53.2%	0.3%	53.2%	0.1%	5.5%	3.3%
4		500	50	67.2%	0.2%	67.2%	0.0%	−0.1%	−0.1%
5			500	64.7%	0.2%	64.8%	0.0%	0.7%	0.1%
6			5000	62.7%	0.3%	62.7%	0.0%	2.5%	−2.2%
7		1000	50	68.9%	0.1%	68.9%	0.0%	0.0%	0.0%
8			500	67.5%	0.0%	67.5%	0.0%	0.2%	−0.1%
9			5000	65.5%	0.2%	65.5%	0.0%	1.4%	−0.2%
10	0.8	100	50	69.3%	0.8%	69.3%	0.1%	4.4%	2.1%
11			500	63.6%	0.4%	63.3%	0.1%	6.8%	3.2%
12			5000	60.6%	0.6%	60.5%	0.1%	9.2%	3.9%
13		500	50	78.4%	0.2%	78.4%	0.0%	−0.4%	0.0%
14			500	76.9%	0.1%	76.9%	0.0%	0.4%	−0.3%
15			5000	74.2%	0.3%	74.2%	0.0%	1.4%	−0.5%
16		1000	50	79.1%	0.1%	79.1%	0.0%	0.0%	−0.1%
17			500	78.6%	0.1%	78.6%	0.0%	0.1%	0.0%
18			5000	77.0%	0.1%	77.0%	0.0%	0.6%	−0.2%
19	0.9	100	50	85.7%	0.4%	85.7%	0.1%	1.9%	0.3%
20			500	81.5%	0.6%	81.5%	0.2%	3.2%	2.2%
21			5000	77.3%	0.7%	76.9%	0.1%	4.3%	2.7%
22		500	50	91.2%	0.1%	91.2%	0.0%	−0.1%	−0.1%
23			500	90.6%	0.1%	90.6%	0.0%	0.0%	−0.1%
24			5000	89.2%	0.1%	89.2%	0.0%	0.3%	0.0%
25		1000	50	91.6%	0.0%	91.6%	0.0%	−0.1%	0.0%
26			500	91.2%	0.0%	91.2%	0.0%	0.1%	−0.1%
27			5000	90.6%	0.0%	90.6%	0.0%	0.2%	0.1%

† This bias is calculated by subtracting the external AUC from the AUC reported by internal CV procedure that was used for selecting the tuning parameter λ. For each scenario, 30 replicated datasets were simulated, and the median and MAD bias of internal CV AUC were summarized over 30 replicated datasets.

‡ This bias is calculated by subtracting the external AUC from the AUC estimated by the outside 5-fold CV wrapper on MCglmnet procedure with s = 50.

**Table 2.** Distribution of number of variables selected in signature. ADNI proteomics data.

Method	Min.	1st Qu.	Median	Mean	3rd Qu.	Max.
glmnet	3	3	10	16.15	29	56
MCglmnet (s = 50)	3	9	10	11.25	14	18

## Data Availability

ADNI data used in illustration is publicly available [8]. Software for implementing our proposed MCglmnet procedure that includes the outside cross-validation wrapper is available via our R package at the following link: https://web.stanford.edu/~lutian/Software. HTML, accessed on 11 January 2023.
